# Association of HLA-A alleles with periodontitis in people living with HIV: A case control study

**DOI:** 10.21203/rs.3.rs-7485455/v1

**Published:** 2025-09-25

**Authors:** Oumo David, Munabi Ian.G, Ochieng Joseph, Erisa Mwaka, Buwembo William

**Affiliations:** Makerere University College of Health Sciences; Makerere University College of Health Sciences; Makerere University College of Health Sciences; Makerere University College of Health Sciences; Makerere University College of Health Sciences

**Keywords:** Periodontitis, HLA, Oxford Nanopore, HIV

## Abstract

**Background:**

Periodontal disease (PD) is a common chronic inflammatory condition that progresses severely in people living with HIV (PLWH). Although human leukocyte antigen (HLA) class I molecules, including HLA-A alleles, are key to immune function, their role in PD susceptibility among PLWH is poorly understood.

**Objective:**

To identify the most frequent HLA-A alleles associated with PD in PLWH and determine those independently linked to PD status.

**Methods:**

A case-control genetic association study was conducted using buffy coat samples from 156 HIV-positive individuals enrolled at the MJAP-ISS Clinic. DNA was extracted, amplified via PCR, and sequenced using the Oxford Nanopore MinION platform. Due to sequencing throughput limitations, only 64 samples yielded usable sequence data. Allele frequencies were compared between PD cases and periodontally healthy controls. Statistical analyses included logistic regression, and ROC curve analysis.

**Results:**

Thirteen HLA-A alleles were identified. HLA-A11 was the most common allele among PD cases (48.4%) compared to controls (7.8%). A11 showed a significant association with PD (adjusted odds ratio [AOR] = 12.8; 95% CI: 3.36–61.6; p < 0.001). A sex-stratified analysis showed that the association was significant among females (AOR = 14.3), but not in males. Other alleles, such as A02 and A03 were not significantly associated. The model combining A11 and sex achieved the best performance (AUC = 0.867).

**Conclusion:**

HLA-A11 is significantly associated with PD in PLWH, especially among females, suggesting a gene–sex interaction. Further studies are needed to explore the immunological basis and validate findings across diverse populations.

Clinical trial Number: Not applicable

## Background

Periodontitis is a progressive inflammatory disease associated with the accumulation of dental plaque (biofilm) and the gradual destruction of tooth-supporting structures like the periodontal ligament and alveolar bone, leading to tooth loss when left untreated [1]. Globally, it affects about 743 million people, making it the sixth most prevalent health condition with a worldwide prevalence of approximately 10.8% [2]. Estimates suggest that between 50–70% of adults experience periodontal disease at some point in their lives [3, 4], and its severity increases with age, affecting up to 60% of adults over 65 years [5]. The burden of periodontitis is notably high in sub-Saharan Africa, where systemic and socio-behavioral factors such as poverty, limited access to dental care, poor oral hygiene, and chronic systemic diseases play a significant role in its prevalence and progression [6]. In Uganda, recent studies report a strikingly high frequency of periodontal disease, with a prevalence of 85.2% among individuals living with diabetes mellitus, especially those with lower educational attainment [7]. Despite the growing concern, there remains limited research focused on the prevalence, risk factors, and genetic contributors to periodontitis within the Ugandan population.

One group particularly vulnerable to periodontal disease is people living with HIV (PLWH). The immunosuppressive nature of HIV infection renders individuals more susceptible to opportunistic infections and inflammatory conditions such as periodontitis. Evidence demonstrates that PLWH are nearly twice as likely to develop severe periodontitis compared to HIV-negative counterparts [8, 9]. Despite the effectiveness of antiretroviral therapy (ART), persistent systemic inflammation and immune dysregulation continue to predispose PLWH to periodontal tissue destruction [10, 11]. A study in the Netherlands found that 66% of HIV-positive participants had severe periodontitis, almost double the prevalence in their HIV-negative counterparts [9]. The pathogenesis is thought to involve heightened levels of pro-inflammatory cytokines (e.g., TNF-α, IL-6, IL-1) and increased infiltration of immune cells like mast cells, neutrophils, and macrophages within gingival tissues [12, 13]. In addition to immunosuppression and behavioral factors, emerging evidence highlights the importance of genetic susceptibility in modulating host responses to periodontal pathogens. Among the most extensively studied genetic contributors are the human leukocyte antigen (HLA) genes, which play a critical role in immune regulation by presenting antigens to T lymphocytes [14, 15]. Genetic variations within the HLA genes, especially at the HLA-A, HLA-B, and HLA-DR loci, have been associated with differences in susceptibility or resistance to numerous infectious and autoimmune diseases, including periodontitis [16, 17].

HLA-A alleles in particular have been implicated in altering the host immune response to periodontal pathogens, potentially influencing the severity and progression of the disease [14]. Specific alleles such as A-2, A-3, A-9, A-11, and A-19 have shown associations with heightened immune responses or vulnerability to periodontal tissue destruction. Within the setting of HIV infection, where immune function is already impaired, the role of HLA-A polymorphisms becomes even more critical. Understanding how these genetic variations influence susceptibility to periodontitis among PLWH could shed light on the complex interplay between host immunity, and systemic disease. However, research on HLA-A allele distribution and its association with periodontal disease in PLWH remains limited, especially in sub-Saharan Africa. In Uganda, there is a paucity of data on the genetic determinants of oral health outcomes in HIV-infected populations. This knowledge gap hinders the development of targeted preventive strategies and personalized treatment approaches that could mitigate the oral health disparities experienced by this vulnerable group. This study, therefore, sought to investigate the association between HLA-A alleles and periodontitis in PLWH in Uganda. By identifying the prevalent HLA-A variants and examining their relationship with periodontal disease, the study aimed to contribute to a deeper understanding of genetic susceptibility and improve oral health management in immunocompromised populations.

## Methods

This was a Case-control study nested in a parent study titled; Oral papillomavirus, microbiota and cancer in people living with HIV(OHPVMC) [18]. A case was defined as an individual living with HIV and diagnosed with periodontitis, while a control referred to a person living with HIV who did not have periodontitis. This study utilized stored samples from the parent study, OHPVMC. The parent study was conducted on PLWH with periodontitis who received care at Makerere University Joint AIDS Program (MJAP)–ISS Clinic. Consent was obtained from participants to collect, store, and use their samples for future research. The participants’ age, gender, occupation, level of education, tobacco use, duration on antiretroviral therapy (ART), and alcohol use data were recorded. The parent study received approval from the Makerere University School of Medicine Research Ethics Committee (SOMREC, REC REF 2022 − 451) and the Uganda National Council for Science and Technology (HS2541ES). It gathered 4,449 samples from October 2022 to October 2023 from the research participants who agreed to take part in the study. There were 2,201 (48.4%) participants with periodontal disease; 1505 with mild PD, 577with moderate PD, and 119 with severe PD.

The sample size for this study was determined using QUANTO, a software tool designed for power and sample size calculation in genetic association studies [19]. Parameters entered into QUANTO included an unmatched case-control design with a gene-only hypothesis, a desired power of 95% (0.95), a two-sided type 1 error rate of 0.05, a log-additive mode of inheritance, an allele frequency of 13% [20, 21], a disease prevalence of 66% [9], and a relative risk (odds ratio) of 2.59 [22]. Based on these inputs, the calculated sample size was 155, to which one additional sample was added to reach an even number of 156, allowing for equal representation of male and female participants.

For this study, a total of 156 samples from people living with HIV (PLWH) on antiretroviral therapy (ART) were selected. Samples were stratified by sex and periodontal disease (PD) status to ensure balanced representation, with equal numbers of male and female participants (78 each). Cases were categorized into three PD severity scores: mild, moderate, and severe, while controls were PLWH without periodontitis. Stratified random sampling using R software ensured even distribution and reduced selection bias. This approach enhanced comparability between groups and supported the integrity of the case-control framework.

Genomic DNA was extracted from buffy coat samples using the Quick-DNA^™^ Miniprep Plus Kit (Zymo Research, California, USA), following the manufacturer’s protocol, which involved lysis, enzymatic digestion with Proteinase K, binding to a silica column, multiple wash steps, and final elution in 100 μL of elution buffer. The extracted DNA was stored at − 20°C for further molecular analysis. After extraction, DNA concentration and purity were assessed using a NanoDrop^™^ spectrophotometer (Thermo Fisher Scientific, California, USA), which measured absorbance at 260 nm to determine concentration and evaluated the A260/A280 ratio to ensure sample purity. Only DNA samples with adequate concentration and acceptable purity levels (~ 1.8 ratio) were advanced to downstream applications like PCR and sequencing. Polymerase Chain Reaction (PCR) was performed to amplify targeted regions of the HLA-A gene from the extracted genomic DNA. Each 50 μL reaction contained a master mix comprising Dream Taq buffer, magnesium chloride, forward and reverse primers, deoxynucleotide triphosphates, Taq DNA polymerase, and 20 ng of DNA template. Specific primers; HLAAF1 (5′-AACTCAGAGCTAAGGAATGATGGCAAAT-3′), HLAAF2 (5′-AACTCAGAGCTATGGAATGATGGTAAAT-3′), and AR1 (5′-ATATAACCATCATCGTGTCCCAAGGTTC-3′) were used to amplify conserved exonic regions (Inqaba Biotec, 2023). The mixture was vortexed and subjected to thermal cycling, as described by Sebastian Johansson [23], which consisted of an initial denaturation at 94°C for 2 minutes, followed by 30 cycles of denaturation at 98°C for 15 seconds, annealing at 60°C for 30 seconds, and extension at 68°C for 4 minutes. The process ended with a final extension at 68°C for 10 minutes. Gel electrophoresis was employed to confirm successful amplification of the HLA-A gene by separating DNA fragments based on size. A 2% agarose gel stained with ethidium bromide was prepared and poured into a casting tray to solidify. PCR products and a 1 Kb DNA ladder were then loaded into the gel, and electrophoresis was run for approximately one hour. Visualization under UV light using the Vilber E-box imaging system (Vilber, Deutschland GmbH, Wieland Strasse 2, Germany) allowed the confirmation of bright bands at expected sizes, indicating that PCR amplification had been successful and the DNA quality was sufficient for further analysis.

Subsequently, PCR products underwent purification using AMPure XP beads to remove unwanted components and prepare the samples for sequencing. The beads were thoroughly mixed and added to each PCR sample to bind DNA fragments. After magnetic separation, ethanol washes were performed to eliminate impurities, followed by air drying to remove residual ethanol. Finally, DNA was eluted with elution buffer and incubated to release clean DNA, which was then ready for downstream processes such as library preparation and sequencing. Barcoding and library preparation were conducted using the Oxford Nanopore Technologies (ONT) Rapid Barcoding Kit to enable multiplex sequencing on a single MinION flow cell. Each PCR product was quantified to ensure an adequate DNA concentration (~ 200 ng in 7.5 μL), after which a unique barcode (RB01–RB12) was added to each sample. The mixture underwent a transposase-mediated reaction at 37°C for 20 minutes, followed by 60°C for 5 minutes, simultaneously fragmenting the DNA and attaching barcode adapters. All barcoded samples were pooled and purified using AMPure XP beads to remove short fragments and excess reagents.

A Rapid Adapter (RAP) was then added to make the library compatible with nanopore sequencing, and the final pooled library was loaded onto the MinION flow cell (R9.4.1) for sequencing. Real-time sequencing was monitored using MinKNOW software. Post-barcoding, DNA quantification was performed using a Nanodrop spectrophotometer to confirm the required concentration and purity. Only high-quality samples were used to prepare the pooled sequencing library. Sequencing output was saved in POD5 format for efficiency and later converted into FASTQ format for downstream quality checks and bioinformatics analysis.

Although DNA extraction, PCR amplification, and barcoding were completed for all 156 samples, only 64 (41%) yielded usable sequencing reads. The remaining 92 samples likely failed due to poor DNA quality, suboptimal amplification, or library preparation issues. Despite the reduced yield, the 64 samples retained for analysis offered a fairly balanced representation across sex and periodontal disease (PD) status, thereby maintaining the integrity of the stratified sampling design. Quality control of the sequence reads was performed using FastQC, and NanoFilt was applied to trim low-quality bases and remove adapters. Cleaned reads were aligned to the HLA reference genome from the IPD-IMGT/HLA database using Minimap2, optimized for long-read data. The alignment, saved in BAM format, allowed the extraction of allele-specific read counts to determine HLA-A allele frequencies. Although the initial sample size of 156 was powered to detect an odds ratio (OR) of 2.59 at 95% power, a post-hoc analysis based on the observed OR for HLA-A11 (13.6) confirmed that the reduced sample size still retained > 95% power for detecting strong associations. Statistical analysis was conducted using R (version 4.5.0). Descriptive statistics summarized demographic and clinical characteristics, while bivariate analyses compared allele distributions across groups using chi-square or Fisher’s exact tests, applying false discovery rate (FDR) correction for multiple comparisons. Multivariable logistic regression models adjusted for covariates like age, sex, ART duration, and alcohol intake to identify independent associations. Interaction effects (HLA-A11 by sex) and subgroup analyses were also performed. Predictive performance of the model was assessed using ROC curves, with the area under the curve (AUC) reported.

### Ethical Consideration:

Ethical approval for this study was granted by the Makerere University School of Biomedical Sciences Research Ethics Committee (SBSREC) under approval number SBS-2024–615, which also issued a waiver of consent. Permission to access and use data and biological samples from the parent study was obtained from the Principal Investigator. All participant-related information was handled with strict confidentiality and was not disclosed to third parties or included in any publications.

## Results

**Figure 1:**
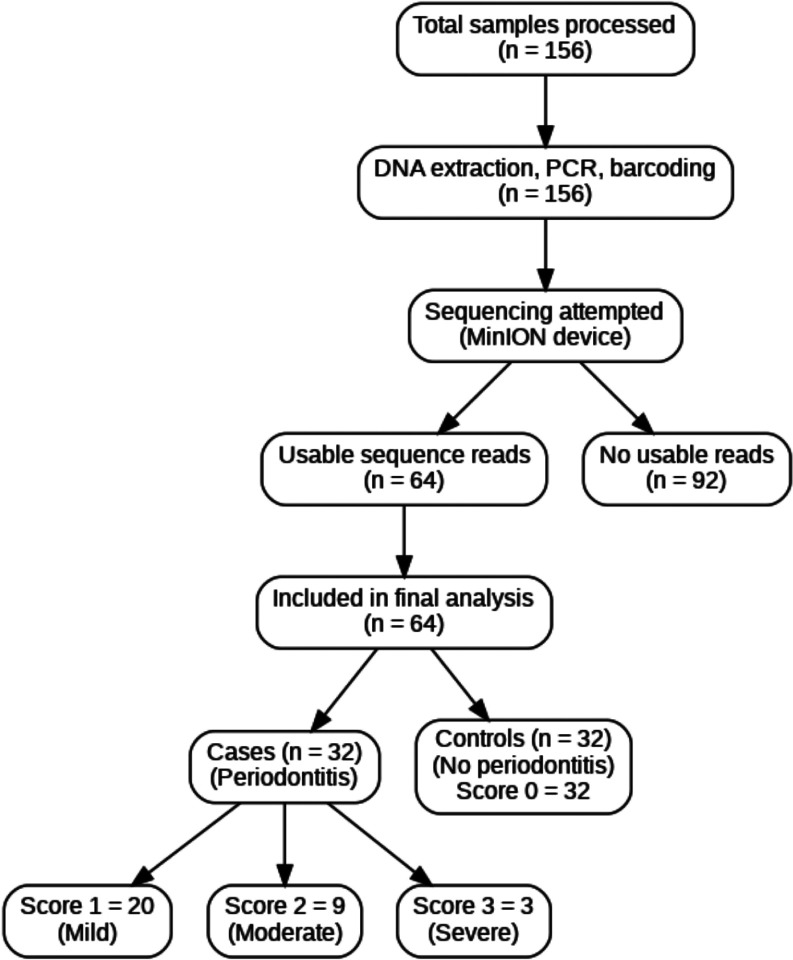
Sample flow diagram

Out of the 156 blood samples processed for genomic DNA extraction and sequencing, only 64 samples (41%) yielded high-quality sequencing reads despite the successful completion of DNA extraction, PCR amplification, and barcoding across all samples. The remaining 92 samples (59%) failed to generate usable data, likely due to technical limitations in library preparation or sequencing. Consequently, downstream bioinformatics and statistical analyses were conducted using the 64 successfully sequenced samples, which maintained a relatively balanced distribution across sex and periodontal disease (PD) status, thereby preserving the stratified sampling framework. These 64 samples that were processed are depicted in Fig. 1. They were predominantly female (64.1%) and ranged in age from 19 to 64 years, with a mean age of 43.95 years (SD = 10.44). All samples were from people living with HIV (PLWH) and had been on antiretroviral therapy (ART) for at least one year, with a median ART duration of 5 years and minimal variation (SD = 0.61). Further demographic and clinical details are summarized in Tables 1.

A total of thirteen distinct HLA-A alleles were identified among the samples in both the periodontitis and healthy control groups. HLA-A11 emerged as the most prevalent allele associated with periodontal disease in people living with HIV (PLWH), appearing 31 times (48.4%) among those with periodontitis compared to only 5 times (7.8%) in the control group. In contrast, other commonly occurring alleles, such as HLA-A02 and HLA-A03, demonstrated a more even distribution between the two groups. Specifically, A02 was detected 9 times (14.1%) in cases and 14 times (21.9%) in controls, while A*03 appeared 8 times (12.5%) in cases and 14 times (21.9%) in controls. A detailed summary of the allele frequencies is presented in Table 2.

A multivariate logistic regression analysis was performed to identify independent factors associated with periodontal disease (PD) among people living with HIV (PLWH). The outcome variable was PD status (coded as 0 for healthy and 1 for PD), and the predictors included selected HLA-A alleles (A11, A03, A02), age, sex, ART duration, and alcohol consumption. The analysis revealed that only the presence of the HLA-A11 allele was significantly associated with increased odds of having PD, with individuals carrying this allele showing 13.6 times higher odds of PD compared to those without it (95% CI: 3.34–72.0, p < 0.001). All other predictors, including other alleles, demographic variables, and lifestyle factors, did not show significant associations in the adjusted model. Detailed results of the regression analysis are presented in Table 3.

In the reduced multivariate logistic regression model, which included only the key HLA-A alleles (A*11, A*03, and A*02), HLA*A11 was significantly associated with periodontal disease (PD) among people living with HIV. Carriers of the A*11 allele had approximately 13-fold increased odds of having PD compared to non-carriers (adjusted odds ratio [AOR] = 12.8; 95% confidence interval [CI]: 3.36–61.6; p = 0.0005). In contrast, the alleles A*03 and A*02 did not show significant associations with PD (AOR = 1.02, 95% CI: 0.25–4.13, p = 0.98; and AOR = 1.35, 95% CI: 0.36–5.33, p = 0.66, respectively).

To further investigate sex-specific associations between HLA-A alleles and periodontal disease (PD), stratified multivariate logistic regression models were conducted separately for male and female participants. Among females, the presence of the HLA-A*11 allele was significantly associated with increased odds of PD (AOR = 14.3, 95% CI: 3.64–76.8, p < 0.001). In contrast, no significant association was observed between A*11 and PD among males (AOR = 0.96, 95% CI: 0.06–10.4, p = 0.973). Other alleles, including A*03 and A*02, showed non-significant trends toward increased PD risk in females and protective effects in males, though these did not reach statistical significance. Age, ART duration, and alcohol intake were not significantly associated with PD in either subgroup.

**Figure 2:**
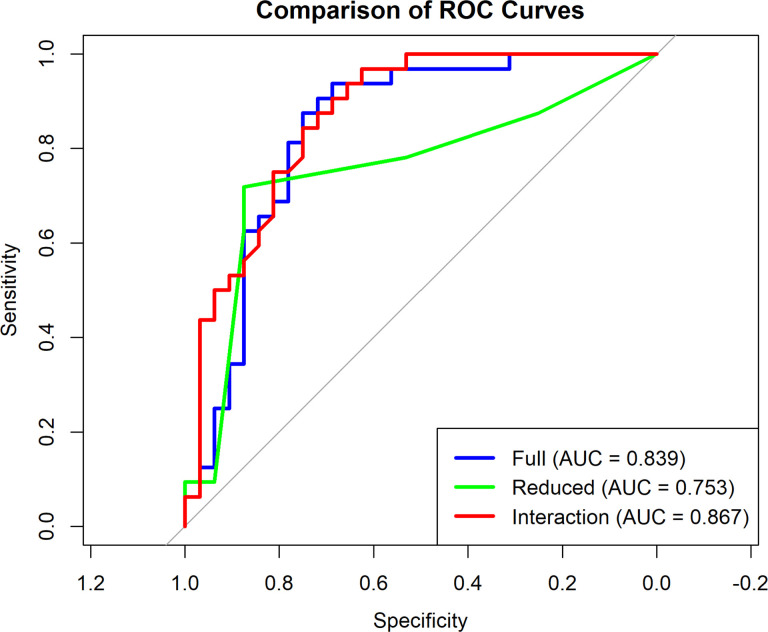
Presents ROC curves for three logistic regression models

To evaluate the predictive performance of the logistic regression models for periodontal disease, receiver operating characteristic (ROC) curves were generated and compared across three models: a full model including all covariates, a reduced model with only HLA-A alleles (A*11, A*03, A*02), and an interaction model that included a sex by A*11 interaction term. The area under the curve (AUC) for the full model was 0.839, while the reduced model had a lower AUC of 0.753, and the interaction model showed the highest AUC of 0.867. Comparison using DeLong’s test revealed that the full model performed better than the reduced model, although the difference was not statistically significant (Z = 1.811, p = 0.070). No significant difference was observed between the full and interaction models (Z = −0.918, p = 0.359). However, the interaction model significantly outperformed the reduced model (Z = −1.976, p = 0.048), suggesting that incorporating the interaction between A*11 and sex improved the model’s predictive accuracy. Figure 2 shows the ROC curve comparing the three logistic regression models.

## Discussion

This study investigated the association between HLA-A alleles and periodontal disease among people living with HIV, with a focus on identifying genetic variants that may confer susceptibility. The significant finding was an association between HLA-A*11 and PD. This allele was present in 48.4% of PD cases compared to 7.8% of healthy controls, and carriers had 13-fold increased odds of PD after adjusting for age, sex, duration on antiretroviral therapy (ART), and alcohol use. Our findings are consistent with previous research implicating HLA-A11 as a susceptibility marker for PD in other populations. A German study similarly reported higher HLA-A11 prevalence in aggressive and rapidly progressive periodontitis, alongside elevated salivary matrix metalloproteinase-8 (aMMP-8) levels, a key mediator of connective tissue destruction [24]. Other studies have associated class I alleles such as HLA-A9, A2, and A26 with PD in different ethnic groups [25, 26]. The present study extends this evidence to the HIV-positive population, where immune dysregulation may exacerbate allele-specific inflammatory responses. HLA-A11 encodes a class I HLA molecule that presents antigenic peptides to CD8+ cytotoxic T lymphocytes [27]. In the context of periodontal infection, altered antigen presentation could drive dysregulated T-cell activation and cytokine secretion, contributing to chronic inflammation and tissue breakdown. Elevated salivary aMMP-8 in HLA-A11 carriers supports a model in which this allele promotes neutrophil hyperactivity and collagen degradation, potentially magnified in the immune-compromised milieu of PLWH.

A novel aspect of this study was the identification of a sex-modified genetic effect: female carriers of HLA-A*11 exhibited markedly higher odds of PD (14-fold) compared to non-carriers, whereas no significant association was observed in males. This pronounced disparity may reflect sex differences in immune regulation, as females typically mount stronger innate and adaptive immune responses [28, 29]. Estrogen-mediated modulation of cytokine production and matrix metalloproteinase activity [30] may further amplify periodontal tissue destruction in genetically susceptible women. Behavioral and psychosocial factors such as stress-related neglect of oral hygiene and hormonal influences on the oral microbiome [31, 32] may also contribute to the observed sex-specific risk amplification.

In contrast, HLA-A*02 and HLA-A*03 were relatively common in the cohort but showed no significant association with PD in either unadjusted or adjusted analyses. This aligns with previous studies reporting neutral or modest effects for these alleles [24, 33]. It is possible that, in the context of HIV-related immune alterations and ART use, these alleles neither significantly protect against nor exacerbate periodontal inflammation. However, subtle immunomodulatory effects cannot be excluded without larger sample sizes and functional immune profiling.

Age was not independently associated with PD in this study, diverging from trends in the general population where older age is linked to greater disease burden [34, 35]. The relatively young mean age of participants (43.95 years) and the overriding effects of HIV-related immunosuppression, systemic inflammation, and ART-induced immune reconstitution may obscure typical age-related patterns [36].

This study demonstrates that HLA-A*11 is significantly associated with increased susceptibility to periodontitis in people living with HIV, highlighting the importance of host genetic factors in the pathogenesis of periodontal disease within this population. By identifying a specific HLA allele linked to heightened risk, our findings contribute to the growing evidence that genetic variation in immune-regulatory loci influences disease progression in HIV-infected individuals. These results underscore the need for further research in larger and more diverse cohorts to confirm this association and to explore its potential role in guiding personalized prevention and management strategies for periodontal disease in PLWH.

## Limitations

This study lacked a non-HIV control group, limiting the ability to determine whether the observed associations are unique to PLWH or reflect general population patterns. Inclusion of matched HIV-negative controls in future research would enhance causal inference and generalizability.

Although 156 samples underwent DNA extraction, PCR amplification, and barcoding, only 64 (41%) produced usable sequencing reads. Resource and financial constraints prevented reprocessing of failed samples, restricting the dataset. While a post hoc power analysis (R version 4.5.0) indicated that the available sample size retained adequate power (95%) to detect strong associations such as the link between HLA-A*11 and periodontitis, reduced power for moderate or small effect sizes may have led to missed associations.

## Conclusion

This study identified HLA-A11 as the dominant allele associated with periodontal disease (PD) among people living with HIV, with a prevalence of 48.4% in affected individuals compared to healthy controls. Other frequently observed alleles included HLA-A02 (14.1%), HLA-A03 (12.5%), HLA-A24 (9.4%), and HLA-A30 (9.4%). Multivariate analysis confirmed a strong, independent association between HLA-A11 and PD, with a particularly pronounced effect among female carriers, suggesting possible sex-specific genetic susceptibility. In contrast, HLA-A02 and HLA-A03 showed no significant associations, indicating a likely neutral role in PD risk in this population. These findings fulfill the study’s objectives and also contribute novel insights into the immunogenetic underpinnings of periodontal disease in the context of HIV, highlighting the potential for genetic markers to inform targeted prevention and personalized care strategies.

## Figures and Tables

**Table 1:** The sociodemographic and clinical characteristics of the participants.

Variable	Category	Frequency (n)	Percentage (%)
**Sex**	**Female**	**41**	**64.10%**
	**Male**	**23**	**35.90%**
**Group**	**Case (Periodontitis)**	**32**	**50.00%**
	**Control (Healthy)**	**32**	**50.00%**
**Periodontal Status**	**Healthy**	**32**	**50.00%**
	**Mild**	**20**	**31.30%**
	**Moderate**	**9**	**14.10%**
	**Severe**	**3**	**4.60%**
**Level of Education**	**Secondary school (O level) completed (S4–S5)**	**64**	**100.00%**
**Occupation**	**Unskilled labor (Shopkeeper, Potter, Maid)**	**22**	**34.40%**
	**No employment / Stay home**	**12**	**18.80%**
	**Self-employed / Business**	**11**	**17.20%**
	**Skilled labor (Carpenters, Tailors, Mechanics)**	**8**	**12.50%**
	**Agriculture (Peasant/Subsistence Farmers)**	**4**	**6.30%**
	**Student**	**3**	**4.70%**
	**Professional / Managerial**	**3**	**4.70%**
	**Sales and services / Clerical**	**1**	**1.60%**

**Table 2:** Distribution of HLA-A Alleles Among Participants with and without Periodontitis

Allele	Healthy (n, %)	PD (n, %)	Total (n)
A*11	**5 (7.8%)**	**31 (48.4%)**	**36**
A*02	**14 (21.9%)**	**9 (14.1%)**	**23**
A*03	**14 (21.9%)**	**8 (12.5%)**	**22**
A*24	**10 (15.6%)**	**6 (9.4%)**	**16**
A*30	**5 (7.8%)**	**6 (9.4%)**	**11**
A*01	**6 (9.4%)**	**1 (1.6%)**	**7**
A*29	**3 (4.7%)**	**2 (3.1%)**	**5**
A*26	**3 (4.7%)**	**0 (0%)**	**3**
A*25	**1 (1.6%)**	**0 (0%)**	**1**
A*32	**1 (1.6%)**	**0 (0%)**	**1**
A*33	**1 (1.6%)**	**0 (0%)**	**1**
A*34	**0 (0%)**	**1 (1.6%)**	**1**
A*80	**1 (1.6%)**	**0 (0%)**	**1**

**Table 3:** Multivariate Logistic Regression Analysis of Predictors Associated with Periodontitis in PLWH

Predictor	Adjusted OR	95% CI	p-value
A*11	**13.6**	**3.34–72.0**	**0.0007**
A*03	**0.99**	**0.22–4.43**	**0.989**
A*02	**1.78**	**0.43–8.11**	**0.436**
**Age**	**1.02**	**0.96–1.08**	**0.514**
**Male Sex**	**3.21**	**0.83–13.8**	**0.099**
**ART Duration**	**1.62**	**0.53–11.7**	**0.518**
**Drinks per Day**	**1.01**	**0.64–1.59**	**0.964**

## Data Availability

The datasets used in this study are freely available: Raw sequencing data: Deposited in NCBI’s Sequence Read Archive (SRA) under accession number [SRP608838] [37]. Processed data and metadata: Figshare. HLA-A allele calls (CSV) [38]. Clinical metadata (Excel) [39]. Barcode-to-sample mapping file (CSV) [40]. All processed files are hosted on Figshare and freely accessible under a CC-BY 4.0 license.

## List of abbreviations

ARTs: Antiretroviral therapy’s
DNA: Deoxyribonucleic Acid
HAART: Highly Active Antiretroviral Therapy
HIV: Human immunodeficiency virus
HLA: Human Leukocyte Antigen
HPV: Human Papilloma Virus
IL: Interleukin
MJAP: Makerere Joint Aids Program
OHPV: Oral Human Papilloma Virus
PCR: Human Papilloma Virus Polymerase Chain Reaction
PD: Periodontal Disease
PLWH: People living with HIV
SBSREC: Makerere University School of Biomedical Sciences Research Ethics Committee
SOMREC: Makerere University School of Medicine Research and Ethics committee
TNF: Tumor Necrosis Factor
